# Climate Change Beliefs and Attitudes Measure: a preliminary psychometric study in a Portuguese community sample

**DOI:** 10.1192/j.eurpsy.2025.1808

**Published:** 2025-08-26

**Authors:** C. Cabaços, A. T. Pereira, M. Carneiro, M. J. Soares, A. I. Araújo, A. Macedo

**Affiliations:** 1 Institute of Psychological Medicine, Faculty of Medicine; 2 Coimbra Institute for Biomedical Imaging and Translational Research, University of Coimbra, Coimbra, Portugal

## Abstract

**Introduction:**

Climate change beliefs influence people’s behaviours such as the adoption of pro-environmental actions, therefore it is crucial to effectively measure them. We developed a short measure (Climate Change Beliefs and Attitudes Measure/CC-BAM), comprising 4 items whose content was adapted from previous studies, namely Loram et al. (2019)’s 8 item climate change denial measure. To the best of our knowledge, there is no instrument that validly assesses these beliefs in the general Portuguese population.

**Objectives:**

To analyse the psychometric properties of the preliminary Portuguese version of the CC-BAM.

**Methods:**

Item content validity was guaranteed by 7 experts. 599 Portuguese adults (mean age=34.40±16.18) answered to the Portuguese preliminary version of CC-BAM (4 items) and to the validated instruments: Climate Change Distress and Impairment Scale/CC-DIS and Pro-environmental behaviour scale/PEBS. Item 1 (“Do you believe in climate change?”) had three possible options of answer: a) “No”, b) “Yes, I believe they are a natural phenomenon” or c) “Yes, I believe they are caused by human activity”. Items 2 (“How convinced are you of your previous answer?”) and 3 (“Do you think climate change is a serious problem?”) were answered on a Likert scale from 0 (“Not much”) to 4 (“Very much”). Item 4 (“How often do you think about climate change?”) was answered on a Likert scale from 0 (“Never”) to 4 (“All the time”). SPSS 29 was used to perform Exploratory Factor Analysis/EFA, descriptive statistics and inferential analysis.

**Results:**

EFA revealed a 1-factor solution with an explained variance of 55.90% confirmed by parallel analysis. Cronbach’s alpha was of .704 (lower if any item excluded) and all items had a corrected item-total correlation >.20. All items correlated with each other (from r=.27 to r=.59) and with the total score (from r=.63 to r=.85, all p<.01). CC-BAM correlated with CC-DIS (r=.51) and PEBS (r=.49, both p<.01). Women had significantly higher mean scores in all items and total CC-BAM. Age correlated with CC-BAM total score (r=.12) and with item 4 (r=.35, all p<.01). 86.5% of participants (n=518) answered to item 1 with option c) (group 1); 12.5% (n=75) answered b) (group 2) and 1% (n=6) responded a) (group 3). Kruskal-Wallis test revealed that those 3 groups significantly differed in mean/median scores of items 2, 3 and 4 and of CC-DIS and PEBS.

**Conclusions:**

The preliminary version of CC-BAM shows adequate psychometric properties, however more studies are needed. Although caution must be taken in the interpretation of this preliminary results, this study provides a novel, internally consistent and short (easily implementable) measure of climate change beliefs.

**Disclosure of Interest:**

None Declared

